# How Does Therapy Harm? A Model of Adverse Process Using Task Analysis in the Meta-Synthesis of Service Users' Experience

**DOI:** 10.3389/fpsyg.2019.00347

**Published:** 2019-03-13

**Authors:** Joe Curran, Glenys D. Parry, Gillian E. Hardy, Jennifer Darling, Ann-Marie Mason, Eleni Chambers

**Affiliations:** ^1^Sheffield Health and Social Care NHS Foundation Trust, Sheffield, United Kingdom; ^2^School of Health and Related Research, University of Sheffield, Sheffield, United Kingdom; ^3^Clinical Psychology Unit, Department of Psychology, University of Sheffield, Sheffield, United Kingdom; ^4^Pennine Care NHS Foundation Trust, Ashton-under-Lyne, United Kingdom; ^5^School of Nursing and Midwifery, University of Sheffield, Sheffield, United Kingdom

**Keywords:** psychotherapy harm, patient safety, negative effects, adverse effects, qualitative systematic review, task analysis

## Abstract

**Background:** Despite repeated discussion of treatment safety, there remains little quantitative research directly addressing the potential of therapy to harm. In contrast, there are numerous sources of qualitative evidence on clients' negative experience of psychotherapy, which they report as harmful.

**Objective:** To derive a model of process factors potentially leading to negative or harmful effects of therapy, from the clients' perspective, based on a systematic narrative synthesis of evidence on negative experiences and effects of psychotherapy from (a) qualitative research findings and (b) participants' testimony.

**Method:** We adapted Greenberg (2007) task analysis as a discovery-oriented method for the systematic synthesis of qualitative research and service user testimony. A rational model of adverse processes in psychotherapy was empirically refined in two separate analyses, which were then compared and incorporated into a rational-empirical model. This was then validated against an independent qualitative study of negative effects.

**Results:** Over 90% of the themes in the rational-empirical model were supported in the validation study. Contextual issues, such as lack of cultural validity and therapy options together with unmet client expectations fed into negative therapeutic processes (e.g., unresolved alliance ruptures). These involved a range of unhelpful therapist behaviors (e.g., rigidity, over-control, lack of knowledge) associated with clients feeling disempowered, silenced, or devalued. These were coupled with issues of power and blame.

**Conclusions:** Task analysis can be adapted to extract meaning from large quantities of qualitative data, in different formats. The service user perspective reveals there are potentially harmful factors at each stage of the therapy journey which require remedial action. Implications of these findings for practice improvement are discussed.

## Introduction

Psychotherapy outcomes are not always positive. Approximately 40–60% of patients do not reach a recovery criterion (Fisher and Durham, 1999; Gyani et al., 2013; HSCIS, 2018) and between 5 and 8.2% have a negative outcome, with worse mental health at the end of therapy than at intake (Barkham et al., 2001; Hansen et al., 2002). Estimates vary because of measurement and population differences. However, there is an important difference between an unsuccessful therapy and a harmful one. Clinical deterioration can be caused by many factors external to the therapy, and failure to benefit from therapy does not imply harm. Negative effects of therapy are common, may be short-lived, and emotionally distressing experience may be an intrinsic part of good therapy (Schermuly-Haupt et al., 2018). Rozental et al. (2019) found that 50.9% of 564 clients in low intensity CBT reported some degree of adverse experience during therapy on the Negative Effects Questionnaire (NEQ). In contrast, in a survey of 14,587 British patients receiving National Health Service psychotherapy, 5% reported “lasting bad effects” of therapy (Crawford et al., 2016). Although this is a much smaller proportion, it represents a large number of patients who report that therapy has been, to some extent, harmful.

Although the broad topic of negative outcomes has been extensively discussed, empirical research on patient safety, directly examining the causes and prevention of harm, is not well established. Because harm (defined here as enduring negative effects directly caused by therapy) is relatively rare, and not amenable to experimental manipulation, such research is difficult. Randomized controlled trials in psychotherapy can monitor adverse events during treatment and could usefully report deterioration rates alongside overall weighted mean differences (Parry et al., 2016) but neither of these methods can directly investigate causes of harm.

Another strategy is to draw on qualitative evidence from patients' reported experience of adverse process and outcome in therapy. In support of this, a report from selected psychotherapy researchers in this field (Rozental et al., 2018) suggested that whilst awareness of negative effects has increased, there remain many unresolved issues. One consensus recommendation to address this was to pursue qualitative methods. Although individual qualitative studies are often small and idiosyncratic, there are sufficient published to enable narrative synthesis of their results. In addition, there are many sources of patient testimony in the “gray” literature and online.

Methods for meta-analysis and thematic synthesis of qualitative evidence are available which provide comprehensive description of a phenomenon and an assessment of the influence of the method of investigation on findings (Thomas and Harden, 2008; Timulak, 2009). Yet they may not in themselves yield a testable process model of the mechanisms by which patient experience is linked to lasting negative effects. To address this directly, we adapted the psychotherapy research method of task analysis (Rice and Greenberg, 1984) to derive and refine such a model.

Task analysis in psychotherapy research was developed by Rice and Greenberg (1984) as an intensive observational method in psychotherapy process research, sensitive to context and based on identifying and describing key change events. An *event* was defined in terms of a patient-therapist interactional sequence with a beginning, a working through process and an end point. In these events, the psychotherapy patient was seen as an active agent engaged in the task of trying to resolve their problem. Identification of key change events requires theoretical understanding and clinical experience and is therefore undertaken by clinician-scientists rather than naïve observers. There are two phases to the method; the discovery phase and the validation phase.

In the discovery phase, a rational model of the process under study is constructed after making the cognitive map of the investigators as explicit as possible and describing the task environment; the wider intervention context. The rational model pulls together the investigators' understanding of how the process unfolds and is a hypothesized possible task performance. This is followed by the empirical task analysis, which is based on a rigorous observation of actual psychotherapy process followed by a form of qualitative content analysis describing a sequence of phenomena that unfold over time. When the first empirical model has been delineated, it is compared to the rational model and used to corroborate, modify or even falsify the rational model. The modified model is then used in a reiterative process of empirical-rational comparison with a new case, until no further discoveries are made (model saturation). The final rational-empirical model completes the discovery phase. The validation phase investigates how well the rational-empirical model describes task resolution and ideally, as a final but less often completed step, tests the extent to which the process predicts therapy outcome.

In this study, we depart from the fundamental purpose of task analysis in analyzing text rather than verbatim therapy process, but we retain the essential logic of the discovery phase of the analytic method. The process under analysis is the course of bad or harmful therapy, with events in the *patient's* experience as the focus of study, although therapist factors are also considered because they are crucial to the task environment. The investigators' cognitive map and the context of poor therapy contribute toward development of the rational model, followed by empirical observation of process reported in (a) qualitative research and in (b) patient testimony. Then we make rational-empirical comparisons to derive two separate models using reiterative sampling of best examples, followed by comparison between them and a final combined rational-empirical model. We finally undertake a partial validation by a structured comparison of the new model against data from an independently-conducted qualitative study.

The aims of this study are to derive a model of process factors potentially leading to negative or harmful effects of therapy, from the patient's perspective, based on a systematic narrative synthesis of evidence on negative experiences and effects of psychotherapy from (a) qualitative research findings and (b) patients' testimony, using task analytic methods.

## Methods

### Overview of Method

Using the principles of task analytic method described above, we adopted the following research strategy:

- A rational model of adverse processes in therapy leading to negative outcomes was developed by a group of psychotherapists, psychotherapy researchers and service users.
- This initial model was then used to inform strategy and keywords for two literature searches: (i) qualitative research reports and (ii) service user reported experiences.
- Data extraction from qualitative research reports of patient experience meeting inclusion criteria was based on themes and categories derived by authors of the original studies, where available. Where no such results were presented, free text of the original studies' interpretation of the respondents' experiences was used.
- Data extraction listed therapy processes, adverse effects, and any reported direct relationships between adverse processes and adverse effects.
- In addition, data on the broader context associated with adverse processes and therapist factors were extracted, using the themes reported in the original studies' analysis of therapists' experiences.
- Service user testimony was obtained from blogs, discussion boards, book chapters, and articles. Data extraction was from patients' verbatim reports of adverse processes and adverse effects of psychotherapy, and any reported direct causal relationships between adverse processes and adverse effects.
- All data categories were coded by initially comparing and matching them to processes in the rational model. If no match was apparent, the original study authors' theme or testimony unit was retained and categorized as “Adverse Process not in Rational Model” for later analysis.
- Two separate rational-empirical comparisons were made, one using qualitative research evidence and the other using service user testimony. In each, the rational model was successively amended and refined to incorporate the empirical coding and categories rejected by the empirical comparison were removed.
- The two rational-empirical models were compared and finally combined into a single rational-empirical model of causal processes for harm.
- The final model from the discovery phase was tested against independent findings from a more recent qualitative study.

**Figure 1:**
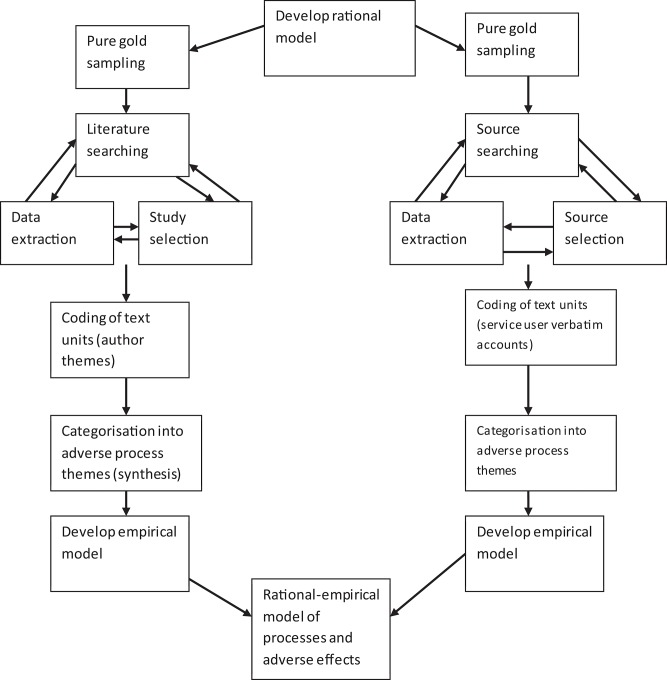
Overview of task analysis.

An overview of the process is shown in Figure 1.

### Development of Rational Model

The “expert” consensus was developed by the first and second authors working with five others; the group consisted of service users, clinical psychologists, counselors, psychotherapists and researchers (some participants having more than one role). The psychotherapy process was broken down into key stages by JC and GP, based on their understanding of psychotherapy process research (e.g., Howard et al., 1993; Schaap et al., 1993). The focus of the model (the “task environment”) was specified as “psychotherapy and counseling." This allowed for the specific inclusion of bona fide psychotherapies, and justified the exclusion of descriptions of psychotherapy, such as equine therapy, that were not considered so.

The group collaboratively constructed a rational model of adverse processes in psychotherapy they considered would lead to adverse effects. They first reflected individually on their experience of psychotherapy and of research findings, in order to develop a list of therapy related events that have led, or could lead, to a therapy causing adverse effects in the client. Detailed discussion of these stages and processes resulted in agreement on eight essential stages and contextual areas (Domains) and 46 adverse processes that were then constructed into a provisional phase model of adverse processes. This was then circulated to the group members for comment, clarification, amendment and agreement. A researcher external to the group (GH) subsequently reviewed this working model to ensure clarity and the expert group then confirmed this final model. After consensus was reached, the rational model of adverse processes was confirmed.

**Figure 2:**
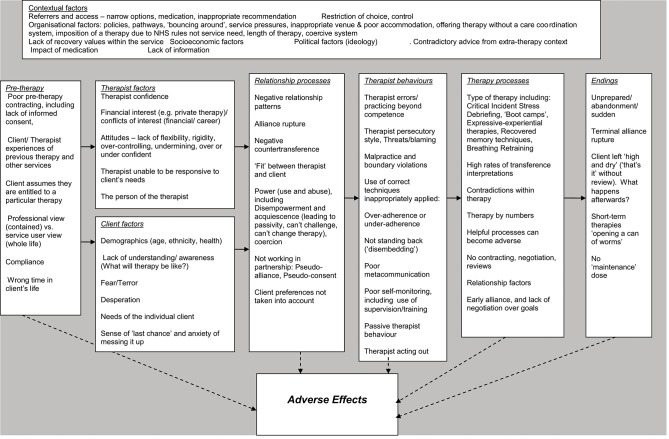
Rational model of adverse processes in psychotherapy.

The Rational model comprised eight Domains (in bold) that were associated with an adverse effect (see Figure 2). The first Domain, **Contextual factors**, contained six themes relating to the setting of therapy, (*Referral and access to service, Organizational factors, Socio-economic factors, Political factors, Lack of information*, and *Impact of medication*). The model then considered a second Domain, **Pre-therapy factors** (*Poor pre-therapy contracting, Experiences of previous therapy, Clients' sense of entitlement, Service is focused on symptoms rather than client as a person, Client too compliant*, and *Wrong time in client's life*). In addition, characteristics that clients and therapists brought to therapy were considered: **Therapist factors** *(Confidence, Financial interest, Attitudes*, and *Person of the therapist)* and **Client factors** (*Demographics, Lack of understanding, Fear, Desperation*, and *Sense of last chance*). These Domains impacted on **Relationship processes** *(Negative relationship patterns, Negative countertransference, Poor fit between client and therapist, Power, Pseudo alliance*, and *Client preferences not taken into account)*, **Therapist behaviors** *(Therapist errors, Therapist persecutory style, Malpractice, Inappropriately applying techniques, Not standing back, Poor meta-communication, Poor self-monitoring, Passive therapist*, and *Therapist acting out*) and **Therapy processes (***Types of therapy, High rates of transference interpretations, Contradictions within therapy, Therapist not responsive to individual client needs, Helpful processes becoming adverse, No contracting***)**. These processes and behaviors finally impacted on therapy **Endings (***Unprepared, Terminal alliance rupture, Short term therapies opening a “can of worms*,” *Client left high and dry*, and *No maintenance dose***)**. All of these Domains are linked to adverse effects.

### Search and Sampling Strategies

Search strategy for qualitative research used the following sources:

- MEDLINE via OvidSP (1946–2011)
- Embase via Ovid SP (1974–2011)
- CINAHL via EBSCO (1981–2011)
- PsycINFO via OvidSP (1967–2011)

A combination of free-text and thesaurus searching was used. Full details of search terms used are available from the authors. Published methodological search filters to limit study type (qualitative) were used where available. Studies were limited to adult participants and those published in the English language. No other search filters were used. Reference sections of included studies were scrutinized for additional potential includes, as were reference lists from relevant reviews and contact with key authors.

In contrast to more general systematic reviews, the intention of the literature search was to look for the best available sources of qualitative research that would facilitate the task analysis. In this context this was determined to be the most clearly observed and described accounts of therapy processes and their consequences, as experienced by patients in psychotherapy. The key data that we sought from qualitative studies were original study author-derived themes, categories or free-text that described a process that participants had experienced as adverse or harmful. These were drawn from the original study authors' qualitative analysis of the research participants' experiences of psychotherapy. Verbatim reports from research participants were extracted only for verification purposes.

The “pure-gold” purposive sampling strategy described by Greenberg (2007) required the researchers to use the following inclusion criteria to identify research which:

- Explores participants' experiences of therapy/counseling
- Reports adverse process and adverse effects
- Yields themes, categories or free-text
- Are the best examples of detailed, thorough and rich data of adverse process and adverse effect in their purest form (i.e., included participants' cognitive, affective, and behavioral experiences in temporal sequence as part of a clearly described therapy event).

The same sampling strategy and definition of adverse processes and effects was used for service user testimony, drawing on the following sources:

- Mental health organizations and websites
- Therapist associations, societies and websites
- Survivor/user groups and websites
- Key book publications
- Blogs and comments on blogs
- Anti-therapy groups and websites
- Newspaper websites
- Law firms
- Journal articles

Google search engine was used to locate Internet sources. Search methods used key search terms within websites, using the “find” function on individual web pages, manually browsing websites, manually searching index and reference lists, key search terms in various combinations, Amazon books online “similar items” function.

Inclusion criteria for service user testimony were

- First person account of experience of psychotherapy
- Detailed description of adverse effect, adverse process, and their relationship

Studies were selected which reported adverse processes and adverse effects in greatest detail and depth. In line with the reiterative nature of task analysis, further studies were selected which provided additional clarification of the nature of the adverse processes, adverse effects and their relationship. Reports of helpful effects of psychotherapy were also sampled, to discriminate the precise nature of the phenomena of interest (Greenberg, 2007), and are described more fully below.

A sample of 32 research studies with data on adverse process and adverse effects was obtained. A sample of 26 studies on the helpful effects of psychotherapy was obtained to aid discrimination (some of these were the same as they covered both helpful and hindering factors).

A sample of 27 sources of service user testimony reporting adverse processes was obtained. A further 16 accounts of helpful therapy were used to inform the discrimination of adverse effects.

Details of referenced sources on which the data extraction was based are given in (Supplementary Data Sheet S1).

### Data Extraction and Quality Appraisal

For qualitative research papers, data on the publication, research method, type of psychotherapy, the phase of therapy, specific adverse process and adverse effects of studies were extracted by one of two researchers, who then compared their results for the sample overall to establish consistency. The quality of the studies was examined using a scale derived from the UK Critical Appraisal Skills Programme (CASP, 2001), and poor quality studies excluded from the sample. Poor quality studies were judged to be those that on this scale did not demonstrate rigorous use of qualitative methods of data collection and data analysis in producing their findings and/or produced unclear statements of their findings (negative ratings on CASP items 1, 2, 8, and 9).

For service user testimony, the data extracted centered on first person accounts of psychotherapy, as well as contextual features of the events and the account. Quality of the reporting of the testimony was completed using a checklist informed by one developed by the Joanna Briggs Institute (2008).

### Derivation of Empirical Categories—Qualitative Research Papers

The first stage in extracting empirical categories was to identify an adverse process marker. This needed to have all of the following features; (a) Be a description of a therapy process, technique, therapist behavior or contextual factor; (b) Be derived by the researchers/authors of qualitative research studies; (c) Be based on an analysis of first-hand accounts of psychotherapy service users' experiences of psychotherapy; (d) Be negatively evaluated (implicitly or explicitly).

Then adverse effects were identified, which needed to be as a consequence of the task marker, experienced directly by the research participant and negatively evaluated (implicitly or explicitly). Codes were applied to the extracted data with reference to the processes identified in the rational model. Where the study authors' themes/service user testimonies and the rational model processes were considered to match (or be synonymous) the rational model term was applied. If the process did not correspond to any rational model code, the study authors' themes were retained for evaluation and synthesis later. For service user testimonies codes were applied to the textual data units. After adverse effects, adverse processes and relationships between them had been identified, the process was repeated for “helpful” factors to aid discrimination.

### Derivation of Empirical Themes—Service User Testimony

The extracted data from testimony were explored and coded, either using the rational model, or where the data did not appear in the rational model, according to the researchers' understanding of the service users' experience, consulting with a service user member of the project steering group. Descriptions/categories and themes coded as adverse effects were recorded and brought together using thematic analysis using the methods described by Braun and Clarke (2006). The resulting categories were constructed into an empirical model of service user experiences of adverse processes of psychotherapy. This resulted in the specification of key themes across several areas, and an overarching theme. A matrix of regularities in relationships between specific adverse processes, (or themes) and adverse effects, where they existed, was constructed.

The contribution of each research paper and service user testimony is provided in  Supplementary Tables S1,  S2, from which two empirical models were developed (available from the authors).

### Rational-Empirical Comparisons and Development of Combined Rational-Empirical Model

The synthesis of the research findings involved construction of a rational-empirical model of adverse processes which incorporated evidence from both empirical models. Two researchers independently reviewed all of the coding for each adverse process reported at each phase of therapy and developed initial ideas for ways of describing the key themes that uniquely distinguish adverse processes in psychotherapy. In order to ensure that the themes were exhaustive, each was applied to every segment of coded data extracted from the selected studies, including the additional category “adverse processes not in the rational model.” These were also applied to the helpful processes to explore whether the adverse process theme was “confirmed,” or whether some contextual consideration applied (for example the impact of therapist self-disclosure varied according to context).

Empirical themes from both research and testimony were successively compared with the descriptions suggested in the rational model, which was refined, modified and extended, adapting the rational model to fit the empirical data. The themes were placed into the model, and further overarching themes derived to account for regularities in adverse processes across stages of therapy.

### Validation Phase

The validation phase usually involves looking at whether the model discriminates between therapy events, such as unresolved and resolved moments in therapy. This comparative method is problematic when considering a whole therapy experience; we therefore adapted this step to include a comparison with a thematic analysis of risk factors for negative experiences of therapy that was developed by the same research group in parallel but with different members undertaking it, blind to the task analysis. Therefore, the results used for validation purposes are entirely independent of the task analysis study. The risk factors in the validation study were developed using the thematic analysis of therapist and patient interviews and questionnaires (see Hardy et al., 2017 for details). The validation process involved three of the authors (JC, GP, and GH) separately comparing the themes of the rational-empirical model to the themes from the qualitative study, noting similarities and differences. Agreement was then reached through discussion, noting which task analytic themes were present in or absent from the thematic analysis.

## Results

The final synthesized rational-empirical model is described below. As before, the Domains (overarching themes) in the Synthesized Model are given in **bold**, and the subordinate themes in *italics*.

**Figure 3:**
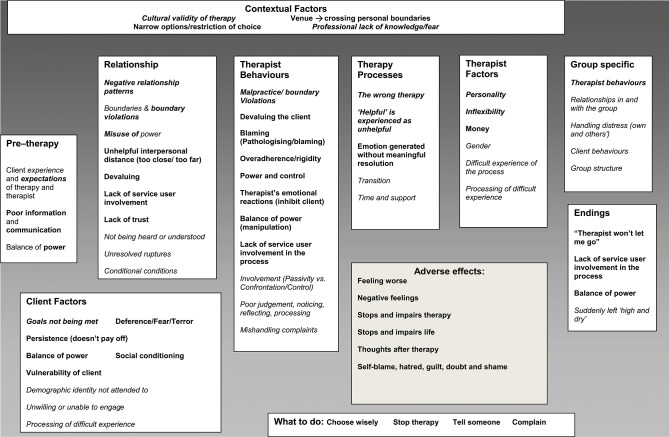
Final synthesized rational-empirical model of adverse effects Bold italic = service user testimony and qualitative research; Bold = Service user testimony only; Italic only = qualitative research only.

The final synthesized rational-empirical model contained 51 themes subsumed under the eight Domains that were identified in the rational model of adverse processes, plus two additional Domains, **What to do** and **Adverse effects**. Nineteen of the subordinate themes were part of the original rational model (these are indicated in Figure 3) and were confirmed in either service user testimonies (*Venue, Narrow options, Poor information, Deference, Money, Blaming, Over adherence*), qualitative research (*Demographic identity not attended to*, and *Suddenly left high and dry*) or both (*Cultural validity of therapy, Professional lack of knowledge, Negative relationship patterns, Misuse of power, Goals not being met, The wrong therapy, Helpful experienced as unhelpful, Malpractice, Personality*, and *Money*). The remaining themes came from either or both of the empirical models but not the Rational Model (see Figure 3).

The final model includes the following themes that have been linked to adverse effects.

### Contextual Factors

These themes include the *Cultural validity of therapy*, which refers to the ways in which therapy and therapists are represented and understood, as suggested by the quote:

We've all been told that this baloney somehow is on the same par with medical services. They've been trained and validated by prestigious institutions. Much of what we watch and read tell us these are serious, qualified, responsible people who will improve our lives if we follow their program (Service User Testimony 1).

The theme *Narrow options/Restriction of Choice* refers to organizational and social factors restricting access to therapy, for example:

Participants in both studies highlighted a range of deficits in conventional services that left them expressing feelings of desperation and powerlessness in a system that appeared to undermine access to effective care (Bee et al., 2010, p. 1310),

Some survivors also seemed to believe that living in certain areas affected their access to services (Chouliara et al., 2011, p.146).

*Professionals' lack of knowledge/fear* was applied to data where therapists' attitudes and emotions were identified as impediment to therapy.

The tendency for health professionals to address symptoms rather than causes led to what many respondents believed was an over-emphasis on a medical model of care and a sole reliance on pharmacological treatments (Bee et al., 2010, p. 1310).

### Pre-therapy Factors

The theme *Client experience and expectations* was developed from clients' experiences of previous therapy (good and bad), and covers client expectations on the nature and structure of therapy and their own role in the process.

The description of a previous therapeutic alliance as “strong” or ‘not strong” were both related to the experience of rupture events in therapy as was a similar episode having occurred before (Coutinho et al., 2010, p. 532).

Negative feelings seemed to occur because of the clients' feelings that their expectations for therapist behavior were breached (Rhodes et al., 1994, p. 480).

“I entered therapy having little idea…what I was getting into” (Patient testimony 6).

### Relationship Factors

Several relationship factors were identified. An important theme that was present in both sets of literature were the derived and directly experienced *Negative patterns in therapy relationships* that were described in several ways:

Experience of an impersonal therapist (Poulsen et al., 2010, p. 487),

“Perceived therapist detachment, and therapist perceived as a threatening and shame-inducing audience” (Grafanaki and McLeod, 1999, p. 297),

Distant and Rigid Therapeutic Relationships (Grunebaum, 1986, p. 170) It really did replicate the experience of having an emotionally abusive parent (Service user testimony 3).

The *Misuse of power* theme refers to the ways in which people felt disempowered in the relationship, as indicated in:

At the same time, he felt pushed by the therapist to pursue a treatment goal that he did not share and by which he felt restricted (Qureshi, 2007, p. 473).

So long as there was a payment and revelations were not mutual, the therapist always had huge power over me, the troubled client (Service User Testimony 3, p. 25).

Clients reported *Not being heard or understood* and *Conditional conditions* refers to the impact of what might be considered the standard, typical or core conditions of therapy may be experienced adversely in some contexts, as suggested by the following:

Negotiating Distance: A sense of professional caring is needed, or the therapist is experienced as too distant, defensive, or un-attuned to clients' emotions. However, caring is too intense if the therapist is experienced as jealous, controlling, or pitying (Levitt et al., 2006, p. 320).

Ruptures that were maintained or not attended to also were seen as leading to adverse effects.

### Client Factors

The theme *Goals not being met* was developed from themes evident in both sets of literature:

All of the patients experienced a conflict between a wish for more simple, functional help in contrast to the intensive therapy they had been given (Wilson and Sperlinger, 2004, p. 227).

So it's like a lottery, only you can either gain big, lose big or land anywhere in between (Service User Testimony 5).

The theme *Vulnerability of clients* was present only in the patient testimony and refers to the reported experience of seeking therapy at particularly vulnerable times, exemplified by:

Particularly after my divorce I felt unattractive and unwanted. I wished to be seen as a viable woman who was worthy of love. I desperately needed to know if Dr. A could see me in such a light. Not to act on it but just to know that he could see those qualities in me (Service user testimony 6).

Although lack of attention to *Demographic identity* of the client could be related to therapist factors or behavior it is in this overarching theme to emphasize the importance of this theme to the client, where failure to address issues such as race, spirituality, or culture led to adverse events.

### Therapy Processes

Certain processes relating to the therapy that clients went through were associated with adverse experiences. Being in the *Wrong therapy* was developed from clients' descriptions that they did not agree with the techniques or model of therapy. More subtly, typical therapy processes can have both helpful and unhelpful effects, for instance Grafanaki and McLeod (1999) analysis of qualitative interviews from clients in experiential psychotherapy identified a theme of “Negotiating a New Story Line” as both helpful and hindering to therapy:

In helpful events, this new story line was perceived as empowering and emancipating. By contrast, in some hindering events, the new story line was regarded as threatening, painful, or untimely (p. 298).

### Therapist Factors

Amongst the several therapist factors the client's perception of the therapist's *Personal characteristics* and or personality adversely affects the therapy process for example:

Therapists described by their patients and having great difficulty dealing with their patients in ordinary human ways and often in a cold or *Inflexible* manner (Grunebaum, 1986).

When I went to therapy, I was looking in large part for a role model, someone who set a good example. What I found was quite the opposite. I so often thought, “I don't want to be like this person; they don't exhibit the values I'd like to live by.” But after the first one, I felt helpless and kept trying to look for help. I didn't know where else to turn (Service User Testimony 1).

A further therapist factor, *Experience of the process/processing of difficult experience* describes clients' experiences of when their therapists appeared not be able to help them process their experience, as in:

None of the patients in the (Emotionally Seductive) group thought that their therapists had helped them sufficiently to work on their feelings that had been aroused (Grunebaum, 1986).

### Unhelpful Therapist Behaviors

Beyond the suggested specific characteristics of therapists, themes around how therapists behaved in ways that led to adverse events were developed. These behaviors included clearly unethical behaviors, captured in the *Malpractice/boundary violations* theme:

As well as writing secretly, we began texting. Some of his texts became very sexually explicit (Service User Testimony 13, p. 125).

It was hindering when the counselor was perceived as pushing his/her agenda onto the client. For example, the client may have felt pressured into participating in certain exercises, engaging in non-sexual touch, remembering past experiences, disclosing the abuse to others, talking about certain topics, or engaging in some behavior outside of the therapy setting for which she did not feel ready. She viewed the counselor as being controlling, rigid, and violating or minimizing her boundaries, and she may even have felt re-abused (Koehn, 2007, p. 47).

They also related to less overt, but still problematic and aversive behaviors. For example, therapists were sometimes seen as *Devaluing* or *Blaming* the client. Therapists were also reported to be too confrontational or characterized as too passive, vague and silent. These behaviors were related to the theme *Involvement*:

It was noted that the therapist did good work but exercised too much control over the direction it took (Service User Testimony 8).

### Endings

The ending of therapy, including where clients choose to end therapy unilaterally or when they felt *Suddenly left high and dry*, and the ways in which it was handled and processed contributed to the overall experience of therapy (Knox et al., 2011). This was characterized by:

No expression of termination related emotion, No review of therapy or client growth, Unplanned termination and No discussion of post-termination plan (Knox et al., 2011).

### What to Do

Four specific themes were developed from the service user testimony in which people reporting adverse effects had provided accounts of actions they had taken to address or resolve these consequences, and were therefore encouraging other to do the same. One suggestion is to *Choose wisely*:

I would say ask for recommendations if you can, and if the person's not right for you, say so, and ask if there's someone else you can see (Service User Testimony 9).

Other suggestions are to *Stop Therapy*, or *Tell someone*:

“I did not do all of this alone. I am lucky to have had a good support network. My husband has been a safe haven of love and support. I have had mental health care providers who understand how to help victims of trauma and sexual abuse. Through TELL and Advocateweb, I have found other victims and professionals willing to share their experiences, thus breaking my feelings of isolation and of being different” (Service User Testimony 10).

This theme also includes the client telling the therapist about their experiences of therapy.

The final suggestion was to *Complain*:

Writing a complaint helped me put the blame where it belongs. My therapist was entirely responsible for what had happened between us. I had done nothing wrong by holding him accountable for his actions (Service User Testimony 11).

### Adverse Effects

This Domain was derived from the qualitative literature. All themes (except one, *No return on investment*) were observed in both the qualitative literature and patient testimonies and included *Feeling worse, Negative feelings, Stops, and impairs life* (patients), *Stops* and *impairs therapy* (qualitative literature), and *Thoughts after therapy*. These themes were evidenced by the strong negative feelings expressed by patients:

I was confused about the nature of our relationship and this confusion resulted in a profound trauma that I am still trying to heal (Service User Testimony 12).

These feelings interfered with therapy:

Impeding involvement—feelings of vulnerability led to desire to disengage (Audeta and Everall, 2010)

and were often long lasting:

Therapy has always tended to reduce my experience of life to monochrome (Service User Testimony 3).

Patients also described feelings of *Self-Blame, Hatred, Doubt, Guilt, and Shame*.

Although most of the Domains identified in the rational model were confirmed in both the qualitative and service user literature, the themes described above often came from the empirical models. The Domain **Therapist** behaviors contained the highest number of themes present in both the rational and one or both of the empirical models (5/10); all other Domains contained at the most two of the rational model themes.

### Validation

Fifty-eight themes from the task analytic model were examined in terms of whether they matched themes from the validation study (Supplementary Table S3). Of the 58 themes coded, 53 matched themes in the validation study (24 were fully matched independently, 29 were partially matched and agreed by consensus). Only 5 remained unmatched. Overall, the task analysis yielded more finely grained themes than the validation study, but overall agreement was acceptable, with 91% of themes matched.

Three themes present in the task analysis were not found in the validation study: the negative therapeutic relationship pattern where an earlier relationship is re-enacted in therapy (transference and counter-transference), the theme on what clients can do to prevent or escape from negative experiences, and a range of difficulties over ending therapy. From the client's point of view, ending could be premature, abrupt, and emotionally unmanageable or conversely, therapy could be difficult to escape from, or to end against the therapist's advice.

### Helpful Processes

Consistent with the method of task analysis, each adverse process theme was contrasted and compared with data, themes or descriptions of helpful processes, using the within-study data for those studies that had examined both adverse and helpful processes. For example, the identification of the “Experience of an impersonal therapist” as an adverse process Poulsen et al. (2010) contributed to the *Negative Relationship Patterns* theme, with the further observation that “the therapist's acceptance of them as people as well as their needs and feelings had been helpful” (op. cit) providing some clarification on the importance of a validating interpersonal process. This was particularly important for processes that become more or less adverse according to context, such as the **Therapy Process “***Helpful is Experienced as Unhelpful”* where the impact of choice on the experience of trauma-focused work affected the participants' experience of the therapy process:

Trauma focused work was largely seen as challenging by some survivors and professionals alike. The challenges by survivors centered mainly on choosing appropriate timing and depth of such work, which may differ for each survivor. Being prepared for the process and being given the option to opt out when it feels too much were important caveats emphasized by survivors (Chouliara et al., 2011, pp. 140–141.)

The refinement of the adverse process themes from the service user testimony involved a similar process of comparison. For example, within the *Negative Relationship Patterns* theme, patients reported relationship patterns which were helpful; these helpful processes were absent in the adverse accounts:

Someone who I feel has the time for me and knows where I'm coming from someone who I feel I can relate to and understands me, being able to face up to painful aspects of myself and memories with support forming a relationship, albeit with a therapist, where I feel safe (Patient testimony 15).

## Discussion

### Methodology

The use of a task analysis paradigm to synthesize two types of qualitative evidence about adverse effects of psychological therapies is innovative. We believe this study demonstrates that it is a feasible and productive method. However, it can be argued that other qualitative systematic review techniques would serve this purpose just as well, for example, realist synthesis (Pawson, 2002). Dixon-Woods et al. (2006) have demonstrated that every stage of such a review process, from asking the review question through to searching for and sampling the evidence, appraising the evidence and producing a synthesis, challenges the frame of conventional systematic review methodology. They conclude that “attempts to impose dominant views about the appropriate means of conducting reviews of qualitative research should be resisted so that innovation can be fostered” (p. 27). It is in this spirit that we used task analysis, as we considered it particularly well-suited to the systematic integration of both qualitative research findings and patients' testimony. In common with realist synthesis, it uses iterative and heterogeneous processes to produce a review of evidence, and, as an interpretive review, uses theoretically derived sampling in a complex field. However, in task analysis these processes are fully explicit and the method is transparent and reproducible rather than opaque and idiosyncratic.

This study has methodological limitations. The literature search preceded the lengthy process of empirical refinement, which preceded the study used as validation, and so is not contemporary. However, there is no reason to believe that people's experiences of therapy have fundamentally altered during this time period; indeed more recent reports confirm that very similar issues continue to be raised (Werbart et al., 2015; Radcliffe et al., 2018). Our verification results were encouraging, although we did not proceed to the final stage of verification, which would require testing whether the model can distinguish between beneficial and adverse therapies in a new, prospective study.

### Findings

The findings of this study bring into sharp focus the experience of service users throughout their therapy journey, demonstrating the multi-causal nature of adverse effects, including service level parameters, patient/client expectations, therapist competence, attitudes, values and behaviors and client vulnerability to disempowerment. Each of these factors has the capacity to influence the others.

The findings suggest that contextual issues, such as lack of cultural validity and limited therapy options, together with unmet client expectations, fed into negative therapeutic processes. Examples of negative process include unresolved alliance ruptures and client disengagement. These involved a range of unhelpful therapist behaviors, such as rigidity, over-control, boundary violations and lack of knowledge, which in turn were associated with clients feeling disempowered, silenced, or devalued. From the service user's point of view, these were coupled with issues of misuse of power and being blamed.

To a surprising extent, many of the themes in the rational model failed to find empirical evidence in their support from the qualitative research sample or the service user testimony. Whilst this may be attributable to the selected sample, it does emphasize the difference in views between professionals, researchers, and clients about adverse process and effects. We found a similar disparity in the views of therapists and clients in a UK survey of their experiences of failed therapies. Patients generally reported their negative experiences as more harmful, whereas therapists with failed therapies rated them as less harmful for their patients (Hardy et al., 2017), although those surveyed were not describing the same therapies. A discrepancy between the views of professionals and their patients or clients is not unique to psychotherapists, and has long been noted in other disciplines (Robinson, 1978).

The service user perspective reveals there are potentially harmful factors at each stage of the therapy journey, rather than simply negative reactions to therapy itself, which require remedial action. There are several implications of this for practice. First is the importance of methods for ensuring the client's voice is enabled to be heard, so that the therapist-client relationship is not enacted within a closed system. This involves the wider system within which therapy is offered, so that client expectations, cultural validity and therapy choices are actively managed prior to therapy starting. The principle of informed consent requires that risks as well as potential benefits of therapy are clearly explained before therapy starts, and there should be explicit guidelines for both therapist and client on how they can address the problems outlined here.

There is a balance to be struck between protecting the framework of the therapy relationship so that it remains safe, confidential and well-boundaried, whilst allowing and empowering the client to find support, if it deteriorates into a negative, potentially harmful state. Suitable methods might be routine consultation with clients (independent of the therapist) on how therapy is progressing, providing clients with pre-therapy information explaining what to expect in therapy and how to know if therapy is causing harm. This could also give details of who to contact if therapy is going badly, and emphasizing that a change of therapist may be necessary in these circumstances. Any of these policy initiatives would need evaluation.

Another important area of practice improvement concerns the training, accreditation and supervision of competence in therapists, all of which could be improved. Currently there is little education in therapy trainings on the potential for harm, the prevalence of negative effects, the importance of informed consent which explains risks as well as benefits, and developing skills in noticing the signs of a negative process and knowing how to address them. Accreditation is usually offered on the basis of completing a course of study and supervised practice rather than on monitored outcomes including negative outcomes. Although many psychotherapy courses routinely use audio or video recordings of sessions in supervision and appraisal, in others supervision is only based on the therapist's account of their client's presentation, the session process and the therapist's feelings and difficulties. When a therapist is out of touch with the client's feelings or behaving unethically, they are unlikely to reveal this in supervision (Ladany et al., 1996). For this reason, direct or indirect observation of practice is always necessary.

Our findings also have implications for research. We must distinguish between those methods which study “objective” negative effects, such as clinical deterioration on outcome measures, and those which focus on the patient's own experience and view of whether the therapy was damaging for them. They are different phenomena. Researchers need to be careful to distinguish between lasting negative effects of therapy (harm) and more transient negative experiences (sometime called “side effects”) which may or may not result in harm. In addition, there is a danger in labeling negative therapy process as a “side effect,” implying an unwanted but inevitable part of a technically correct treatment procedure. This medical terminology does not capture the co-constructed nature of the therapeutic relationship and the negative interactional patterns that both therapists and clients are drawn into.

We do not yet have a complete understanding of what causes harm and how to prevent it. The divergence between patients' and therapists' understanding of negative effects should be acknowledged and is a neglected topic of research in this field. For example, in line with Rozental et al. (2018) recommendation of more qualitative research, understanding the similarities and differences between therapists' and patient's perception of the same therapy, in a sample of failed therapies, would be illuminating.

Finally, findings in this field are now robust enough to support intervention studies. Using implementation science methods, a fruitful line of services research would evaluate the impact of introducing organizational systems of harm reduction.

## Author Contributions

JC: conducted the data searches, data extraction and the full task analysis, contributed to the validation phase and co-drafted the paper; GP: designed the study, contributed to rational model development, contributed to the validation phase and co-drafted the paper; GH: conducted the validation study, contributed to the validation phase, and co-drafted the paper; JD: coded and derived themes in the analysis of the qualitative literature and patient testimony; A-MM: coded and derived themes in the analysis of the qualitative literature and patient testimony; EC: contributed to rational model development, assisted with data searching and designed the data extraction forms (particularly for the patient testimony).

### Conflict of Interest Statement

The authors declare that the research was conducted in the absence of any commercial or financial relationships that could be construed as a potential conflict of interest.
