# Supplementary material for: How Does Therapy Harm? A Model of Adverse Process Using Task Analysis in the Meta-Synthesis of Service Users' Experience
Source: Front Psychol. 2019 Mar 13;10:347. doi: 10.3389/fpsyg.2019.00347 (PMC6425860; doi:10.3389/fpsyg.2019.00347)
Supplement: Supplementary file 1 [file Data_Sheet_1.docx]

**Supplementary Data Sheet S1**

**Sources for the qualitative research review empirical synthesis. a= adverse and helpful effects, b = adverse effects only, c = helpful effects only**

Adams, J. (2008) Chapter 6. In Sarah Richardson & Melanie Cunningham (Eds) Broken Boundaries – Stories of Betrayal in Relationships of Care. Ch. 6 123-130 London:Witness (b)

Audeta, C. T. & Everall, R. D. (2010) Therapist self-disclosure and the therapeutic relationship: a phenomenological study from the client perspective. British Journal of Guidance and Counselling, 38, 327-342 (a)

Binder, P., Holdersen, H. & Hostmark, N. (2009) Why did I change when I went to therapy? A qualitative analysis of former patients’ conceptions of successful psychotherapy. *Counselling and Psychotherapy Research, 9*, 250-256 (c)

Bevan, A., Oldfield, V. B. and Salkovskis, P. (2010) A qualitative study of the acceptability of an intensive format for the delivery of cognitive-behavioural therapy for obsessive-compulsive disorder, *British Journal of Clinical Psychology*, *49*, 173–191 (a)

Bowman, L. & Fine, M. (2000): Client Perceptions of Couples Therapy: Helpful and Unhelpful Aspects, *The American Journal of Family Therapy*, *28*, 295-310 (a)

Bury, C., Raval, H, & Lyons, L. (2007) Young people’s experiences of individual psychoanalytic psychotherapy. *Psychology and Psychotherapy: Theory, Research and Practice*, *80*, 79–96 (b)

Chouliara Z., Karatzias, T., Scott-Brien, G., Macdonald, A., MacArthur, J., & Frazer, N. (2011) Talking therapy services for adult survivors of Childhood Sexual Abuse (CSA) in Scotland: perspectives of service users and professionals. *Journal of Child Sexual Abuse, 20*, 128-156 (a)

Clarkson, P. & Nippoda, Y. (1997) The experienced influence or effect of cultural/racism issues on the practice of counselling psychology – a qualitative study of one multicultural training organization. *Counselling Psychology Quarterly;* *10*, 415-437 (a)

Coutinho, J., Ribeiro, E., Hill, C. & Safran, J. (2011): Therapists' and clients' experiences of alliance ruptures: A qualitative study. Psychotherapy Research, 21, 525-540 (a)

Cragun, C.L., & Friedlander, M. L. (2012) Experiences of Christian Clients in Secular Psychotherapy: A Mixed-Methods Investigation. Journal of Counseling Psychology, 59, 379–39 (a)

Cunningham, A. J., Phillips, C., Stephen, J. & Edmonds C. (2002) Fighting for life: a qualitative analysis of the process of psychotherapy-assisted self-help in patients with metastatic cancer, Integrative Cancer Therapies, 1, 146 – 161 (a)

Estrada, A. & Holmes, J. M. (1999) Couples’ perceptions of effective and ineffective ingredients of marital therapy. Journal of Sex and Marital Therapy, 25,151-162 (a)

Hare, J. (2016) A silent self. In Y. Bates (ed) Shouldn’t I be feeling better by now. Client views of therapy. Palgrave. (b).

Gehart D. R & Lyle, R. R. (2001) Client experience of gender in therapeutic relationships: an interpretive ethnography, Family Process, 40, 443-458 (a)

Grafanaki, S. & McLeod J. (1999) Narrative processes in the construction of helpful and hindering events in experiential psychotherapy, Psychotherapy Research, 9, 289-303 (a)

Grunebaum, H. (1986). Harmful psychotherapy experience. American Journal of Psychotherapy, 40, 165-176. (b)

Hoffmann, L. L, Gleave, R. L., Burlingame, G. M., Jackson, A. P., (2009) Exploring interactions of improvers and deteriorators in the group therapy process: a qualitative analysis. International Journal of Group Psychotherapy; 59, 179-197 (a)

Hummelen, B., Wilberg, T. & Karterud, S. (2007). Interviews of female patients with Borderline Personality Disorder who dropped out of group psychotherapy. International Journal of Group Psychotherapy, 57, 67- 91 (b)

Israel, T., Gorcheva, R., Burnes, T.R. & Walther W. A. (2008) Helpful and unhelpful therapy experiences of LBGT clients. Psychotherapy Research, 18, 294-305. (a)

Knox, S., Catlin, L., Casper, M. & Schlosser L. Z. (2005): Addressing religion and spirituality in psychotherapy: clients’ perspectives , Psychotherapy Research, 15, 287-303 (a)

Knox, S. Adrians, N., Everson, E., Hess, S., Hill C. & Crook-Lyon (2011) Clients’ perspectives on therapy termination. Psychotherapy Research, 21,154-167 (a)

Koehn, C. V. (2007) Women’s perceptions of power and control in sexual abuse counselling. Journal of Child Sex Abuse, 16, 37- 60 (a)

Levitt, H., Butler, M., & Hill, T. (2006) What clients find helpful in psychotherapy: developing principles for facilitating moment to moment change. Journal of Counseling Psychology, 53, 314-324. (a)

Macdonald, J., Sinason, V. & Hollins, S. (2003) An interview study of people with learning disabilities experience of, and satisfaction with, group analytic therapy. Psychology and Psychotherapy: Theory, Research and Practice, 76, 433-453. (a)

Messari, S. & Hallam, R. (2003) CBT for psychosis: a qualitative analysis of clients’ experiences. British Journal of Clinical Psychology, 42, 171-188. (a)

Nilsson, T., Svensson, M, Sandell, R. & Clinton, D. (2007) Patients’ experiences of change in cognitive –behavioral therapy and psychodynamic therapy: a qualitative comparative study. Psychotherapy Research, 17, 553-566. (a)

Poulsen, S., Lunn, S. & Sandros, C. (2010) Client experience of psychodynamic psychotherapy for bulimia nervosa: an interview study. Psychotherapy Theory, Research, Practice, Training, 47, 469–483 (a)

Qureshi, A. (2007) I was being myself but being an actor too: The experience of a Black male in interracial psychotherapy. Psychology and Psychotherapy: Theory, Research and Practice, 80, 467–479 (a)

Rennie, D. L. (1994) Clients’ deference in psychotherapy. Journal of Counseling Psychology, 41, 427-437. (b)

Rhodes, R. H., Hill, C., Thompson, B. J., & Elliot, R. (1994) Client retrospective recall of resolved and unresolved misunderstanding events. Journal of Counseling Psychology, 41, 473-483 (a)

Roe, D., Dekel, R., Harel, G., Fennig, S. & Fennig, S. (2006a) Clients' feelings during termination of psychodynamically oriented psychotherapy. Bulletin of the Menninger Clinic; 70, 68-81 (a)

Roe, D., Dekel, R., Harel, G., Fennig, S. & Fennig, S. (2006b) Clients’ reasons for terminating psychotherapy: A quantitative and qualitative inquiry. Psychology and Psychotherapy: Theory Research and Practice, 79, 529-538.

Valkonnen, J., Hänninen, V., Lindfors, O. (2011) Outcomes of psychotherapy from the perspective of the users, Psychotherapy Research, 21, 227-240 (a)

Watson, V. C., Cooper, M., McArthur, K., McLeod, J. (2012) Helpful therapeutic processes: Client activities, therapist activities and helpful effects, European Journal of Psychotherapy & Counselling, 14, 77-89 (c)

Watson, J. C. & Rennie, D. L. (1994) Qualitative analysis of clients’ subjective experience of significant moments during the exploration of problematic reactions. Journal of Counseling Psychology, 41, 500-509. (a)

Wilson, M. & Sperlinger, D. (2004) Dropping out or dropping in? Psychoanalytic Psychotherapy, 18, 220-237. (b)
