# Supplementary material for: How Does Therapy Harm? A Model of Adverse Process Using Task Analysis in the Meta-Synthesis of Service Users' Experience
Source: Front Psychol. 2019 Mar 13;10:347. doi: 10.3389/fpsyg.2019.00347 (PMC6425860; doi:10.3389/fpsyg.2019.00347)
Supplement: Supplementary file 2 [file Table_1.docx]

Supplementary Table S1

**Summary table for adverse processes and adverse effects in qualitative research studies**

| **Study** | **N** | **Context** | **Population/ client group** | **Therapy type** | **Data collection** | **Data analysis** | **Adverse processes contribution to empirical model** | **Adverse effects** | **Related**  **Y/N** | **Helpful effects** |
| --- | --- | --- | --- | --- | --- | --- | --- | --- | --- | --- |
| Audeta et al (2010) | 9 | Participants who had completed therapy | Had received individual therapy and experienced therapist self-disclosure | Not available | Face to face interviews | Phenomenological | Therapist self-disclosure | - Role devaluation/reversal  - Role confusion/ role uncertaintly  - Feeling misunderstood  - Feeling overwhelmed | Y | Y |
| Bee at al 2010 – Study 1 | 15 | Qualitative service evaluation of a work-based therapy service | Self-defined anxiety, depression agoraphobia and panic | CBT | Telephone interviews | Thematic analysis | Overemphasis on medical model  Deficits in conventional services  Deficits in conventional services  Satisfaction dependent on perception of therapist’s ongoing availability.  Resistance to remote psychotherapy supervision rooted in rejection of CBT modality (a) | Feelings of desperation and powerlessness | Y |  |
| Bee at al 2010 – Study 2 | 15 | Nested qualitative study within an RCT | Employees absent from work for mental health problems | CBT | Telephone interviews | Thematic analysis |  |  |  |  |
| Bevan et al (2010) | 12 | Participants had completed treatment | OCD | Intensive format for delivery of CBT for OCD | Face-to-face interviews | Thematic analysis | - Perceived quality of therapy  -Ongoing support  -Time between sessions  -Thinking too much  - Stress | Unacceptability and unhelpfulness of the intervention | N | Y |
| Bowman et al. (2000) | 10 (5 couples) | University-based couple and family therapy centre | Not given | Couples Therapy | Face-to-face interviews | Grounded theory and Taylor and Bogdan 1984 | -Unequal treatment of partners  - Therapist talks when client wants to talk  - The term ‘therapy’  - One-hour session is too short  - Not enough bridge between therapy and life | ‘Unhelpful aspects of therapy’ | N | Y |
| Bury et al (2007) | 6 | Young people aged 17-21 | Presenting clinical problems included depression,  eating disorders, self-harm, behavioural difficulties, relationship and emotional  problems | Individual psychoanalytic psychotherapy | Face-to-face interviews | IPA | -Expectations of therapy – fear of being judged  -Expectations appeared to be deeply influenced by images portrayed through the media  - Endings: Feelings of separation and loss  - Power (theme) - sense of powerlessness during some part of their therapeutic experience | Therapy as physically and emotionally exhausting  therapy as a whole was not experienced as helpful: | Y | Y |
| Chouliara et al (2011) | 13 service users (31 professionals) | Adult survivors of CSA in Scotland, UK | Service user survivors of CSA, currently clients of mental health services | Data only available for professionals | Face to face interviews | IPA | - Challenges of using services  - Difficulties of trauma-focussed work  - Contact between appointments  - Continuity and consistency  - Accessibility in acute episodes | Negative aspects of service user experience  ‘Worsening of symptoms’ (re Trauma focussed work) | **Y** | **Y** |
| Clarkson and Nippoda (1997) | 108 | Therapists and trainee counsellors | All experienced personal therapy | Counselling or ‘counselling variants’ | Open-ended questionnaire | ‘Categories of themes’ | Negative statements – race and cultural issues had a negative effect on counselling and psychology  Counselling errors/mistakes  Prejudice/Transference  Feeling de-skilled and other competency issues  Bad and inferior feelings | Feeling deskilled and other competency issues  Counselling errors/mistakes | **N** | **Y** |
| Coutinho at al (2011) | 8 client-therapist dyads | University counselling centre | All clients had a DSM-IV personality disorder | Typically cognitive techniques | Face-to-face interviews | Consensual Qualitative Research | Precipitants of rupture event  Experience of the event  How therapists dealt with the rupture  Similarities between the rupture event and the Client’s personal life | Withdrawal and Confrontation ruptures | **Y** | **N** |
| Cragun and Freidlander (2012) | 11 | Christian clients in secular psychotherapy | Not stated | “Secular therapy” | Face-to-face interviews | Consensual Qualitative Research | Preferred therapist to understand their religious beliefs  Wishing therapist would have asked about faith more  Limited focus on spiritual and religious issues in therapy  Therapists mishandled religious spiritual issues | Feeling judged, disrespected and not listened to | Y | Y |
| Cunningham et al 2002 | 22 | Process of psychotherapy assisted self-help in patients with metastatic Ca | Metastatic cancer/ cholangiocarcinoma/ multiple myeloma | Group psychotherapy and psychoeducation | Face-to-face interviews | Grounded method using Schatzman’s dimensional analysis | Low involvement – Rejecters, Skeptical/Detached, Blocked by personal issues  Variation in involvement in self-help | Less improvement | Y | Y |
| Cunningham and Goyton 2005 (Diss) | 6 | Premature termination through therapist licence revoked | Not stated |  | Face-to-face interviews | Hermeneutic framework | *Endings* | Grief  Transference  Enraged  Reproduction of trauma  Trust  Authority  Symptoms – panic attacks and depression  Autonomy issues  Regression  Retroactively alter  Shame/self-blame  Betrayal  Isolation/Withdrawal  Disillusionment | Y | N |
| Estrada and Holmes 1999 | 15 couples | Experiences of couples therapy | : (a) the couple had been  married for at least 3 years, (b) the couple had a child younger than 12  years of age, (c) marital dissatisfaction and the possibility of divorce were  identi. ed as major problems by at least onemember of the couple during  intake, (d) both partners were available and consented to full participation  in treatment, (e) both partners expressed a desire to improve the  relationship and avoid divorce if possible, and (f ) neither partner met  the criteria for a DSM-IV diagnosis of Major Affective or Psychotic Disorder.  Couples were given a 15% reduction in clinical fees per session for  their participation in the study. | Marital Therapy | Brief Therapy Interview | Data coding and sorting for themes (?Thematic analysis) | 1. Wasting time in therapy.  2. Therapist techniques are unclear.  3. Therapist does not help in resolving issues.  4. Therapist is not acting empathically  .  5. Being unfocused in therapy. | *Poor Outcome – Dyadic Adjustment Scale* | N | Y |
| Gehart and Lyle 2001 | 15 | Gender in the therapeutic relationship | Experienced therapy with both female and male therapists | Couples therapy  Family treatment  Individual | Face-to-face interviews | Hermenuetics | Unhelpful and helpful effects of therapist gender | Unhelpful effects | Y | Y |
| Grafanaki and McLeod 1999 | 6 clients, 6 therapists | Helpful and hindering events in experiential psychotherapy | Variety of personal concerns | Person-centred experiential therapy | Face-to-face interviews | Structured Narrative Analysis of Psychotherapy Segments | Therapist as audience, Negotiating a new story line, | Unhelpful therapy | N | Y |
| Grunebaum (1986) | 47 | Harmful psychotherapy | Not stated | Psychotherapy or analysis | Telephone interviews | Development of themes | Varieties of harmful therapy experiences  Distant and Rigid Therapeutic Relationships  Emotionally Seductive therapists  Sexual involvement  Multiple involvement in cults | Harmful therapy | N | N |
| Hoffman et al (2009) | 20 | Group therapy improvers and deteriorators | One general therapy group. One group for woman who had experienced sexual abuse | Group psychotherapy | Transcripts and videos of group sessions | Hermeneutic analysis | Deteriorator themes  Early and substantial disclosure  Open praise of the process of group  Expectation of the requirement of sharing deep personal information | Deterioration | Y | Y |
| Hummelen (2007) | 8 service users | Dropouts | Borderline Personality Disorder | Group Psychotherapy | Face to face interviews | Hermeneutic | Group therapy too distressing  Outpatient group was not sufficient  Not able to make use of group therapy  Complicated relationship to the group  Negative aspects of the patient-therapist relationship | Group therapy too distressing  Too much rumination after each group session  Arousal of strong negative feelings during treatment  Lost trust | N | N |
| Israel et al. (2008) | 42 | LBGT | Relationships depression/suicidality, career, sexual orientation/gender identity, anxiety/stress and  family. Others  medical health and mandated therapy  . Other presenting concerns were  adjustment, substance abuse, personal growth, body  image, chronic mental health issues, anger, and self -esteem. | Group Therapy  Individual Therapy  Couple Therapy | Face to face interviews | Ethnographic content analysis | clients feeling  unsafe, disrespected, or uncomfortable  clients felt that  their self-determination was compromised  focusing on sexual orientation/  gender identity inappropriately  therapists judging, invalidating,  or misunderstanding the client  failing  to create a connection with the client and  hospitalizing the client  **was clients experiencing**  **the therapist as cold, disrespectful, disengaged,**  **distant, or uncaring**  were **therapists using interventions that**  **clients found ineffective**  by **therapists imposing their**  **values, judgment, or decisions on clients** |  | N | Y |
| Knox et al (2005) | 12 | Religion/spirituality in therapy | Depression, anxiety, family-of-origin issues, trauma, loss | Counselling | Telephone interviews | Consensual Qualitative Research | Therapist passed judgement/imposed beliefs | Clients felt traumatized, confused, frustrated, stuck, angry or judged  Not satisfied with therapy | N | Y |
| Knox et al (2011) | 12 | Termination of therapy within last three years | anxiety/stress (n = 8), anxiety/stress (8), family and relationship concerns (8), mood disorders (6), abuse (3), loss (3) ,  and other (insight/training, health concerns, ADD). | Psychoanalytic/psychodynamic (n=4), cognitive/CBT (n=2), interpersonal (n=1), client-centred (n=1) | Face-to-face and telephone interviews | Consensual Qualitative Research | Variant discussing and planning of termination | Typically highly upset and devastated  Fear and hesitation about seeking future therapy | N | Y |
| Koehn (2007) | 50 | Adult females who had been sexually abuse in childhood | Sought counselling for sexual abuse treatment, ot depression, anxiety, relationships, sexual dysfunction, loss, work issues, eating disorders, substance abuse fears, hearing voices, sexual assault, self-destructive  behavior, and other types of childhood abuse | Had attended at least one or more individual counselling sessions in adulthood | Face-to-face interviews | Data categorisation | Approach to Power and Control (major category  Sexual interest  Response to criticism | Hindering events  feeling “discouraged,” “defeated,” and “like giving up.”  felt angry and terminated therapy  felt afraid,  uncomfortable, awkward, betrayed, angry, “dirty,” disgusted, and a  deep revulsion–“like throwing up.”  . For some women, these incidents  damaged their trust in men and in counselors. | Y | Y |
| Levitt et al (2006) | 26 | Within one year of completing therapy | concerns such as familial issues, assertiveness, depression, rape, anxiety, anger, attention deficit/hyperactivity disorder, and eating disorders | Ranged across major therapeutic orientations | Face-to-face interviews | Hermeneutic grounded theory | Clients may enter therapy with expectations or fears that work against engagement  Transsforming… from person to client and back again  Transgressions of structure  Too distant or overinvolved  Confrontation  Notetaking | Not worthwhile  Ineffective  Diminishing engagement  Prevented from exploring helpful experiences  Made him feel just like a number | Y | Y |
| MacDonald et al (2003) | 9 | People with learning difficulties experience of group analytic therapy | Men: 4 members of a group for sexual offenders  Women: 5 members of a women’s group | Group psychodynamic psychotherapy | Face-to-face interviews | IPA | *Talking is distressing*  *Other participants’ distress is distressing*  *Therapists are too confrontational*  *Negative reminders*  **Negative aspects of group members**  *Negative patient behaviours*  *Others in group dissimilar*  *Group conflict (women)*  *Other group members absent (women*  *Concrete problems*  Noise outside  Not having tea and biscuits  Sleeping during the group*)*  *Other*  Not enjoying the group | Denying a change had taken place | N | Y |
| Messari and Hallam (2003) | 5 | Client experiences of CBT for Psychosis | All clients had a diagnosis of paranoid schizophrenia | CBT for Psychosis | Face-to-face interviews | Discourse analysis | *CBT participation as compliance with powerful medical establishment*  *Client passivity* | Not helpful or enjoyable | N | Y |
| Nilsson et al (2007) | 32 | Patients’ experiences of therapy | Previously completed therapy. presenting  Psychopathology covered a spectrum of  both neurotic and psychotic problems | CBT or Psychodynamic Therapy | Face to dace interviews | Intensive qualitative analysis. | *the therapist to be*  *intrusive and oppressive, applying a rigid predetermined*  *therapy design, whereas the PDT patient felt*  *her therapist was withdrawn, disengaged, and aloof,*  *not providing the support and guidance that the*  *patient had required and expected. Whereas the PDT patient felt herself left alone and deserted, the* | Dissatisfied  Insufficient change  (CBT) Change too short-term | Y | Y |
| Poulsen et al (2010) | 15 | Individual therapy | Bulimia Nervosa (DSM-IV) | Psychodynamic Psychotherapy | Face-to-face interviews | Grounded Theory & Giorgi’s phenomenological approach to meaning condensation | *Hindering factors;*  *The Non-directive approach*  *Unstructured setting*  *The absence of physical contact*  *The age and appearance of the therapist*  *Experiencing the therapist as impersonal/disinterested*  *Dependency in relation to the therapist*  *Experiencing the therapist as ignorant*  *Feeling that the therapist changed*  *The Therapeutic Frame: Duration of therapy* | Greater reservations towards their therapies.  Improvements made life more difficult | N | Y |
| Qureshi (2007)  0 | 1 | Interracial psychotherapy | Not stated | Jungian | Face-to-face interview | IPA | *Therapist components that contributed to and detracted from the therapy process*  Technique and focus  Therapist attitude  Cultural and racial similarities/dissimilarities  Extra-verbal components  Racial transference | Felt coerced  Feeling he had to be a certain way, that the way he was not‘ok’  he felt invasive | Y | Y |
| Rennie (1994) | 14 | University counselling centre | Not stated | Therapists collectively adhered to person-centred, gestalt, transactional analytic, radical behaviouristic, rational-emotive and eclectic orientations | Observation | Grounded Theory | *Deference* | Clients usually just refrained from being openly agential in their interaction with their therapists  Negative appraisal of the therapist competed with their attention to thr part of their experience being addressed in the discourse with their therapist  At other times, , the inner disturbance lasted throughout the session and even beyond. | N | N |
| Rhodes et al 1994 | 19 | Resolved or unresolved misunderstanding events | Participants were also therapists or therapists in training | Dynamic  Humanistic-dynamic  Humanistic  Eclectiv | Questionnaire | Comprehensive Process Analysis | *Misunderstanding events* | Client had negative feelings towards the therapist  Think about quitting and then quit therapy  Client felt “withheld from” and accordingly distanced herself from the therapist  Client does not discuss decision to terminate | Y | Y |
| Richards and Timulak 2012 | 80 | Helpful or hindering events | College students experiencing  depression | Therapist-delivered or self-administered online CBT for depressions | Helpful Aspects of Therapy form | Interpretive qualitative framework outlined by Elliot and Timulak 2005 | Burden of work  Time/pace  Technical  Treatment content  Form of treatment | Hindering impacts  Self-critical/blaming  Frustrating  Irritating  Confusing  Anxious  Disappointing  Painful insight | N | Y |
| Roe et al,(2006) | 88 | Reasons for terminating psychotherapy | Not given | Psychodynamically oriented psychotherapy (private practice) | Semi-structured postal questionnaire | Open coding 🡪 axial coding | *Relationship*  *Dissatisfaction with the therapist*  *Dissatisfaction with psychotherapy* | Loss of a meaningful relationship  Premature termination and need for further treatment  Hurdles within therapeutic dyad  Therapist’s response  Termination unprocessed  Lack of progress  Disappointment, dissatisfaction and feelings of failure | N | Y |
| Valkonen (2011) | 14 | User perspective of psychotherapy outcome | Longstanding depression causing work dysfunction | Solution Focused Therapy or long-term psychodynamic psychotherapy | Face-to-face interviews pre and post therapy | Hermeneutic circle, including thematic analysis and narrative analysis | *Stagnated Inner Narrative*  *Experience and Expectations* | Remain unchanged  Disappointment  Solutions not found for the acute problems |  | Y |
| Watson and Rennie (1994) | 8 | Client subjective experience during problematic reactions | Moderate to high anxiety in the State SSTAI  Mildy depressed on the BDI  Interpersonal problems related to significant others | Individual psychotherapy | Face to face interviews, systematic evocative unfolding and IPR | Grounded Theory | Negative session momentum   | Hindering the in-session exploration of problematic reactions | N | Y |
| Wilson & Sperlinger (2004) | 12  (6 therapists, 6 clients) | Dropouts | Attending psychological therapy services | Psychoanalytic psychotherapy | Semi-structured interviews | IPA | (Patient data only)  *Approach-Avoidance conflict*  *Conflicting wishes for functional help versus intensive therapy*  *Deatchment from vs. involvement with the therapist*  *Therapy as threat and loss of control*  *Fears of dependence, loss or abandonment* | General discomfort  ‘structure of the therapy, the way it worked unnerving’  Loss of control | Y | Y |

Notes: a) taken from author text
