# Supplementary material for: How Does Therapy Harm? A Model of Adverse Process Using Task Analysis in the Meta-Synthesis of Service Users' Experience
Source: Front Psychol. 2019 Mar 13;10:347. doi: 10.3389/fpsyg.2019.00347 (PMC6425860; doi:10.3389/fpsyg.2019.00347)
Supplement: Supplementary file 3 [file Table_2.docx]

**Supplementary Table S2: Sources of service user testimony**

| **Author** | **Type** | **Source** | **Therapy type** | **Therapy settings** | **Number of contributors** |
| --- | --- | --- | --- | --- | --- |
| Marie Hellewell (2006) | Book chapter | The Client Says Not. In Yvonne Bates (Ed) Shouldn’t I Be Feeling Better By Now? Ch. 6. pp 63-76 Basingstoke: Palgrave Macmillan | Unclear | Individual therapy | 1 |
| Jo Hare (2006) | Book chapter | A Silent Self. In Yvonne Bates (Ed) Shouldn’t I Be Feeling Better by Now? Ch. 2. Pp. 20-31 Basingstoke: Palgrave Macmillan | 1. Group psychotherapy 2. Private psychotherapy Freudian psychoanalyst 3. No details given 4. Cognitive psychologist 5. NHS Psychiatrist 6. Two counsellors    1. Very experienced    2. Co-counselling 7. Primal therapy (briefly) 8. Gestalt 9. NHS Psychotherapist 10. One-to-one inner child sessions   11. Telephone counselling | Various | 1 |
| S.T. (2012) | Essay | http://therapyconsumerguide.com/a-damaging-quest-for-help/ | Psychotherapy | Individual | 1 |
| Marilyn | Essay | Therapy Exploitation Link Line  ://www.therapyabuse.org/t2-keeping-secret.htm | Psychotherapy with a psychiatrist | Individual therapy | 1 |
| Peterson | Blog | Therapy Exploitation Link Line  therapyabuse.org­t2-story-is-diffent.htm | Psychoanalytic psychotherapy | Individual therapy | 1 |
| Sylvia Wilde (2006) | Book chapter | Love in an Estate of Bondage. In Yvonne Bates (Ed) *Shouldn’t I Be Feeling Better by Now?* Ch 1 pp 7- 19. Houndsmills, Basingstoke, Hampshire: Palgrave Macmillan. | 1. Freudian and Kleinia 2. Unclear 3. Jungian 4. Unclear | Individual private therapy (x4) | 1 |
| Adams (2008) | Book chapter | Chapter 5. In Sarah Richardson & Melanie Cunningham (Eds) *Broken Boundaries – Stories of Betrayal in Relationships of Care.* Ch. 5 123-130 London:Witness | Counselling with part-time counsellor (BASRT member) at G+*P surgery | Individual therapy, primary care | 1 |
| Mosaics (2000) | Discussion board | Therapy: Helpful and Harmful. In *alt.support.dissociation* URL: https://groups.google.com/d/topic/alt.support.dissociation/qf2aNZmBhkk/discussion | Unclear | Unclear | 4 |
| Nicole Todd (2009) | Report | Focus on the Facts: My Experience of Writing a Licensing Board Complaint. In *TELL (Therapy Exploitation Link Line]*. URL: <http://therapyabuse.org/t2-board-complaint.htm> | Unclear | Individual therapy | 1 |
| Nicole Todd (2007) | Report | Taking Action: What, Why, and How Far? In *TELL (Therapy Exploitation Link Line).* URL: [www.therapyabuse.org/t2-filing-complaint.htm](http://www.therapyabuse.org/t2-filing-complaint.htm) | Not given | Licensed therapist | 1 |
| EarlGreyDregs (2008) | Blog | Psychology & Mental Health Blog – Session #8 Psych forums  <URL:http://www.psychforums.com/blog/EarlGreyDregs/session_8_> | Psychoanalysis | Individual therapy | 1 |
| Noah Rubinstein (2008) | Webpage and discussion | 50 Warning Signs of Questionable Therapy and Counseling. In *GoodTherapy.org*. URL: <http://www.goodtherapy.org/blog/warning-signs-of-bad-therapy/> | Various | Various | 35 |
| Therapyisacon (2012) | Blog | http://therapyisacon.wordpress.com/author/therapyisacon/ | Psychotherapy | Individual | 1 |
| Dr Greg Hinkle (2012) | Blog | When Help isn’t Helpful: Holding onto Hope after a Bad Therapy Experience. WordPress.com. URL: [www.wordpress.com/2012/...when-help-isnt-helpful-holding-onto-hope-after-a-bad-therapy-experience/](http://www.wordpress.com/2012/...when-help-isnt-helpful-holding-onto-hope-after-a-bad-therapy-experience/) (no longer available) | Unclear | Variious | 5 |
| Anon (2012) | Blog with comments | The seductive allure of the “nice” therapist. In *ED Bites*. URL: ed-bites.blogspot.co.uk [Accessed 18/10/12] [Available at <http://ed-bites.blogspot.com/2012/12/the-seductive-allure-of-nice-therapist.html>] | Unclear | Unclear | 4 |
| Marie Schepsisi (2006) | Book chapter | Bates (2006) | Analytic psychotherapy (Jungian) | Individual private therapy | 1 |
| Anthony Smith (2006) | Book chapter | Bates (2006) | Unclear – composite model within the psychodynamic school * | Individual private therapy | 1 |
| Stella_blues (2006) | Discussion board | Uncommonknowledge  www.uncommonforum.com/viewtopic.php?=21260 | ?Cognitive therapy | Outpatient | 6 |
| Wise Monkey (2011) | Discussion board | PsychForum (Moderator salted_lipstick)  www.psychforums.com/psychotherapy/topic79528 | Unclear | Group and individual | 2 |
| Megan (2007) | Discussion board | Megan (2007) choosing therapy. In *uncommon knowledge*. URL: [www.uncommonforum.com/viewtopic?t=24952](http://www.uncommonforum.com/viewtopic?t=24952) | Psychotherapy  Counseling  CBT | Individual therapy | 2 |
| Megan (2007) | Discussion board | the end of therapy. In *uncommon knowledge*. URL: <http://www.uncommonforum.com/viewtopic?t=25340> | Psychodynamic therapy | Individual therapy | 5 |
| Sweetgurl (2007) | Report/blog | Voices Yahoo  voices.yahoo.com/therapeutic-abuse-experience-abusive-therapist-549696.html?cat=72 | Not given | Individual private therapy | 1 |
| Anon (2004) |  | Relationships with our Therapists. *In Forums at Psych Central* Post no. ####778741. URL: <https://forums.psychcentral.com/psychotherapy/3640-relationships-our-therapists.html> | Various | Various | 18 |
| Marilyn Nowack (2005) | Report | Dark Secrets – from Victim to Survivor. *TELL (Therapy Exploitation Link Line).* <URL:http://www.therapyabuse.org/t2-keeping-secret.htm> | Psychotherapy | Psychiatrist individual outpatient | 1 |
| Disequilibrium1(2010) | Blog and discussion | Bad therapy? A disgruntled ex-psychotherapy client speaks her piece. [Accessed 18/07/12] Available at: <http://disequilibrium1.wordpress.com/2010/10/10/a-disgruntled-ex-psychotherapy-client-speaks-her-piece> | Unclear | Individual outpatient | 11 |
| Heather Bell  (2008) | Blog | In *uncommon knowledge*. URL: [www.uncommonforum.com/viewtopic.php?t=38728](http://www.uncommonforum.com/viewtopic.php?t=38728) | Various | Individual therapy | 9 |
| Kate Webster (2010) | Report | Topics: A Victim’s Statement to the Court.  *TELL (Therapy Exploitation Link Line)* <URL:http://www.therapyabuse.org/t2-statement.htm> | Not given | Individual private therapy | 1 |
| Lost_In_Thought (2012) | Discussion board | Therapy Makes Everything Worse . In *Psych Forum*. URL: [www.forum.psychlinks.ca/therapy-and-therapists/29401-therapy-makes-everything-worse.html](http://www.forum.psychlinks.ca/therapy-and-therapists/29401-therapy-makes-everything-worse.html) | Psychotherapy | Individual | 5 |
