# Supplementary material for: How Does Therapy Harm? A Model of Adverse Process Using Task Analysis in the Meta-Synthesis of Service Users' Experience
Source: Front Psychol. 2019 Mar 13;10:347. doi: 10.3389/fpsyg.2019.00347 (PMC6425860; doi:10.3389/fpsyg.2019.00347)
Supplement: Supplementary file 4 [file Table_3.docx]

**Supplementary Table S3: Validation phase: Task and thematic analysis comparison**

| **Task analysis themes** | **Task analysis theme description** | **Validation study theme** |
| --- | --- | --- |
| 1, Cultural validity of therapy | How therapy is portrayed in culture | Psychosocial context |
| 2. Venue 🡪 crossing personal boundaries | Therapist using home as office. | Boundary management |
| 3. Mishandling of complaints | Concerns raised about previous therapists are not listened to. | Being silenced/ belittled |
| 4. Narrow options/restriction of choice | Organizational and social factors restricting access to therapy | Lack of options; Access and continuity |
| 5. Professional lack of knowledge/fear | Thoughts and feelings that may influence therapy | Therapists working beyond their level of competence |
| 6.. Client experiences and expectations of therapy and therapist | Previous therapy experiences and current expectations | Expectations |
| 7. Poor information and communication | Lack of sufficient detail to give informed consent. | Being silenced/ belittled |
| 8. Balance of power | Little negotiation or agreement over process | Lack of information; Being silenced/ belittled |
| 9. Negative relationship patterns | The relationship experienced as negative | No match |
| 10. Boundaries and boundary violations | Therapist fails to maintain appropriate professional boundaries | Unethical behavior; Boundary management |
| 11. Misuse of power | Which led to clients being disempowered | Being silenced/ belittled |
| 12. Unhelpful interpersonal distance | Too close/ too distant | Therapists’ attitudes. |
| 13. Devaluing | Service user contribution to the process not recognised | Dismissed or blamed |
| 14. Lack of service user involvement | ‘Doing to’ not with the client | Core therapy skills. |
| 15. Lack of trust | Not being able to trust the therapist | Loss of trust |
| 16. Not being heard or understood | Not hearing overt content of clients’ communication | Lack of voice, Being silenced/belittled |
| 17. Unresolved ruptures | Therapeutic relationship rupture not repaired | Lack of voice, Being silenced/belittled |
| 18. Conditional conditions | Core conditions of therapy not used responsively | Core therapy skills |
| 19. Goals not being met | Not getting the help needed | Client’s expectations of service not met |
| 20. Deference | Clients assuming a passive role | Lack of voice |
| 21. Fear | Expressing fear | Pandora’s box; fear of feelings |
| 22. Persistence (doesn’t pay off) | Staying with unproductive therapy | Pressure to perform |
| 23. Balance of power | Therapists having or holding some power | Higher order match: Power and Control |
| 24. Social conditioning | What people have learned about therapy and therapists that put them in a less powerful position | Psychosocial context |
| 25. Vulnerability of client | Clients seeking therapy at emotionally vulnerable times | Psychosocial context |
| 26. Demographic identity not attended | Mishandling of demographic identity of the client | Core therapy skills; Psychosocial context |
| 27. Unwilling or unable to engage | Clients finding it hard to engage | Lack of motivation |
| 28. Processing of difficult experience | Presence of difficult thoughts or feelings in the process of therapy | Pandora’s box; fear of feelings |
| 29. The wrong therapy | Clients disagreeing with the techniques being asked to engage in | Lack of voice |
| 30. Helpful experienced as unhelpful | Normal therapy behavior experienced as unhelpful | Dismissed or blamed |
| 31. Emotion generated without meaningful resolution | Emotions elicited but not processed | Pandora’s box; fear of feelings |
| 32. Transition | Such as moving from one therapist to another | Access and continuity |
| 33. Time and support | Lack of support between sessions or sessions too short | Access and continuity |
| 34. Personality | Clients’ perceptions of therapist | Therapists’ attitudes |
| 35. Inflexibility | Rigid and cold therapist | Therapists’ attitudes |
| 36. Money | Therapist lets in be known they need the money from sessions | Unethical behavior |
| 37. Gender | Specific aspects of reported gender characteristics | No match |
| 38.Malpractice/ boundary violations | Unethical behavior | Unethical behavior |
| 39. Devaluing the client |  | Being silenced/ belittled; Dismissed or blamed |
| 40. Blaming (pathologising) |  | Being silenced/ belittled; Dismissed or blamed |
| 41. Overadherence/rigidity | Lacking flexibility or not individualizing therapy | Core therapy skills; Labeling |
| 42. Power and control | Therapists dominating clients | Higher order theme: Power and Control  Being silenced/ belittled; Lack of voice; Dismissed or blamed; |
| 43. Therapist’s emotional reaction inhibits client | Therapists convey an emotional response that affects the client unhelpfully | Therapists’ attitudes |
| 44. Balance of power (manipulation) |  | Being silenced/ belittled |
| 45. Lack of service user involvement in the process |  | Therapists’ attitudes |
| 46. Involvement | Passivity versus confrontation or control | Therapists working beyond their level of competence |
| 47. Poor judgement, noticing, reflecting, processing | Lack of reflexivity and sensitivity | Core therapy skills |
| 48. Therapist won’t let me go | Client wants to end but remain in therapy as therapist doesn’t agree | no match |
| 49. Lack of service user involvement in the process (ending) | No collaboration over ending | no match |
| 50. Balance of power |  | no match |
| 51. Suddenly left high and dry | Ending unplanned and sudden | no match |
| 52. Feeling worse | Enduring worsening of symptoms | Loss of coping |
| 53. Negative feelings | Feeling sad, upset or unhappy after therapy sessions or the end of therapy | Feelings of failure, Loss of coping |
| 54. Stops and impairs life | Major disruptions to functioning after therapy | Loss of coping |
| 55. Stops and impairs therapy | Impediments to the therapy process, dropping out from therapy | Loss of hope |
| 56. No return on investment | Having put a lot of personal and financial resource not getting anything back in terms of outcome | Regret |
| 57. Thoughts after therapy | Rumination on therapy events | Loss of hope Feelings of failure. Regret |
| 58. Self-blame, hatred, doubt, guilt and shame | Intense negative thoughts about self as a consequence of adverse processes in therapy | Lack of confidence, failure, Loss of hope |
